# 2-Amino-5,7-bis­(4-fluoro­phen­yl)-1′,3′-dimethyl-7,8-dihydro­spiro­[pyrido[2,3-*d*]pyrimidine-6(5*H*),5′-pyrimidine]-2′,4,4′,6′(3*H*,1′*H*,3′*H*,5′*H*)-tetra­one ethanol solvate

**DOI:** 10.1107/S1600536809022946

**Published:** 2009-06-20

**Authors:** Xiao-Tong Zhu, Ge Zhang, Ning Ma

**Affiliations:** a Department of Chemistry, Xuzhou Medical College, Jiangsu 221004, People’s Republic of China; b College of Chemistry and Chemical Engineering, Xuzhou Normal University, Xuzhou 221116, People’s Republic of China

## Abstract

In the mol­ecule of the title compound, C_24_H_20_F_2_N_6_O_4_·C_2_H_5_OH, the pyrimidine ring is oriented at dihedral angles of 42.64 (3) and 62.94 (3)° with respect to the benzene rings, while the dihedral angle between the benzene rings is 74.45 (3)°. The pyridine ring adopts an envelope conformation. In the crystal structure, inter­molecular N—H⋯O and O—H⋯N hydrogen bonds link the mol­ecules into a two-dimensional network, forming *R*_2_^2^(8) ring motifs. π–π contacts between the pyrimidine and benzene rings [centroid–centroid distances = 3.516 (1) and 3.927 (1) Å] may further stabilize the structure.

## Related literature

For bond-length data, see: Allen *et al.* (1987 ▶). For ring-motifs, see: Bernstein *et al.* (1995 ▶).
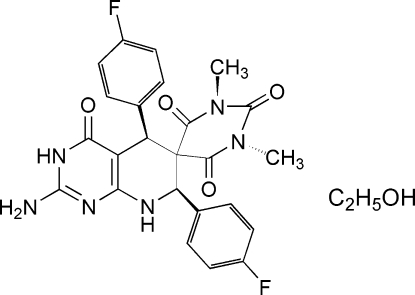


## Experimental

### 

#### Crystal data

C_24_H_20_F_2_N_6_O_4_·C_2_H_6_O
*M* *_r_* = 540.53
Triclinic, 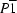

*a* = 9.2189 (15) Å
*b* = 12.5924 (17) Å
*c* = 14.100 (2) Å
α = 64.634 (2)°
β = 81.467 (3)°
γ = 69.027 (2)°
*V* = 1381.0 (4) Å^3^
*Z* = 2
Mo *K*α radiation
μ = 0.10 mm^−1^
*T* = 298 K
0.40 × 0.37 × 0.12 mm

#### Data collection

Bruker SMART CCD area-detector diffractometer
Absorption correction: multi-scan (*SADABS*; Sheldrick, 1996 ▶) *T*_min_ = 0.961, *T*_max_ = 0.988
7223 measured reflections
4773 independent reflections
1979 reflections with *I* > 2σ(*I*)
*R*_int_ = 0.036

#### Refinement

*R*[*F* ^2^ > 2σ(*F* ^2^)] = 0.066
*wR*(*F* ^2^) = 0.120
*S* = 1.00
4773 reflections
352 parameters
H-atom parameters constrained
Δρ_max_ = 0.16 e Å^−3^
Δρ_min_ = −0.18 e Å^−3^

### 

Data collection: *SMART* (Bruker, 1998 ▶); cell refinement: *SAINT* (Bruker, 1999 ▶); data reduction: *SAINT*; program(s) used to solve structure: *SHELXS97* (Sheldrick, 2008 ▶); program(s) used to refine structure: *SHELXL97* (Sheldrick, 2008 ▶); molecular graphics: *SHELXTL* (Sheldrick, 2008 ▶) and *PLATON* (Spek, 2009 ▶); software used to prepare material for publication: *SHELXTL* and *PLATON*.

## Supplementary Material

Crystal structure: contains datablocks global, I. DOI: 10.1107/S1600536809022946/hk2707sup1.cif

Structure factors: contains datablocks I. DOI: 10.1107/S1600536809022946/hk2707Isup2.hkl

Additional supplementary materials:  crystallographic information; 3D view; checkCIF report

## Figures and Tables

**Table 1:** Hydrogen-bond geometry (Å, °)

*D*—H⋯*A*	*D*—H	H⋯*A*	*D*⋯*A*	*D*—H⋯*A*
N3—H3⋯O1^i^	0.86	1.88	2.737 (3)	177
N4—H4*A*⋯O5^ii^	0.86	2.07	2.890 (3)	160
O5—H5⋯N2^iii^	0.82	2.19	2.779 (3)	129

Symmetry codes: (i) 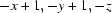; (ii) 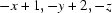; (iii) 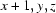.

**Fig. 1.**
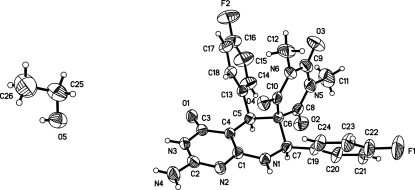
The molecular structure of the title molecule, with the atom-numbering scheme. Displacement ellipsoids are drawn at 30% probability level.

**Fig. 2.**
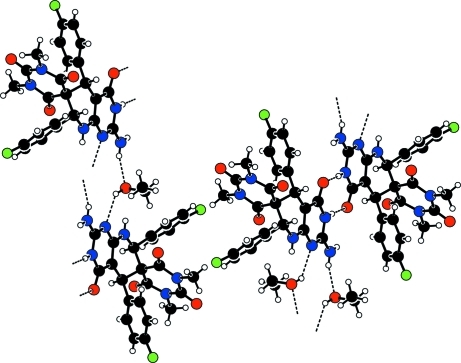
A partial packing diagram for the title compound. Hydrogen bonds are shown as dashed lines.

## supplementary crystallographic information

### Comment

Domino reactions, in an environmentally benign and atom economic fashion, especially considering that certain complex compounds with high diastereoselectivities such as 6-spirosubstituted pyrido[2,3-*d*]pyrimidine, are of great significance and are very effective and attractive. Heterocyclic spirocompounds exhibiting structural rigidity due to conformational restriction are of interest in synthetic organic chemistry. Indeed, the presence of a spirocarbon atom induces a relatively large steric strain and allows thermal, base, acid or photo-promoted rearrangement of these products, yielding new and often unexpected heterocycles. Therefore, the syntheses of these spiral structures were of considerable interest in the pharmaceutical and agrocultural chemistry. We report herein the crystal structure of the title compound.

In the molecule of the title compound (Fig 1), the bond lengths (Allen *et al.*,  1987) and angles are within normal ranges. Rings A (N2/N3//C1-C4), C (N5/N6/C6/C8-C10), D (C13-C18) and E (C19-C24) are, of course, planar. The dihedral angles between them are A/C = 86.54 (3), A/D = 61.88 (3), A/E = 55.57 (3), C/D = 42.64 (3), C/E = 62.94 (3) and D/E = 74.45 (3) °. Ring B (N1/C1/C4-C7) adopts envelope conformation with atom C6 displaced by -0.695 (3) Å from the plane of the other ring atoms.

In the crystal structure, intermolecular N-H···O and O-H···N hydrogen bonds (Table 1) link the molecules into a two-dimensional network forming *R*_2_^2^(8) ring motifs (Bernstein *et al.*, 1995), in which they may be effective in the stabilization of the structure. The π–π contacts between the pyrimidine and phenyl rings, Cg3—Cg4 and Cg3—Cg5, [where Cg3, Cg4 and Cg5 are centroids of the rings C (N5/N6/C6/C8-C10), D (C13-C18) and E (C19-C24), respectively] may further stabilize the structure, with centroid-centroid distances of 3.516 (1) and 3.927 (1) Å, respectively.

### Experimental

The title compound was prepared in vial (10 ml), 2,6-diaminopyrimidine-4(3*H*)-one (126 mg, 1 mmol), 1,3-dimethylbarbituric acid (156 mg, 1 mmol), 4-fluorobenzaldehyde (248 mg, 2 mmol) and water (2.0 ml) were mixed, and then capped. The mixture was irradiated for 7 min at 373 K (initial power 150 W and maximum power 250 W).

### Refinement

H atoms were positioned geometrically, with N-H = O.86 Å (for NH and NH_2_), O-H = 0.82 Å (for OH) and C-H = 0.93, 0.98, 0.97 and 0.96 Å for aromatic, methine, methylene and methyl H, respectively, and constrained to ride on their parent atoms, with U_iso_(H) = xU_eq_(C,N,O), where x = 1.5 for methyl H and OH H and x = 1.2 for all other H atoms.

### Crystal data

**Table:**

C_24_H_20_F_2_N_6_O_4_·C_2_H_6_O	*Z* = 2
*M**_r_* = 540.53	*F*(000) = 564
Triclinic, *P*1	*D*_x_ = 1.300 Mg m^−^^3^
Hall symbol: -P 1	Melting point > 573 K
*a* = 9.2189 (15) Å	Mo *K*α radiation, λ = 0.71073 Å
*b* = 12.5924 (17) Å	Cell parameters from 1085 reflections
*c* = 14.100 (2) Å	θ = 2.5–26.2°
α = 64.634 (2)°	µ = 0.10 mm^−^^1^
β = 81.467 (3)°	*T* = 298 K
γ = 69.027 (2)°	Block, colorless
*V* = 1381.0 (4) Å^3^	0.40 × 0.37 × 0.12 mm

### Data collection

**Table:**

Bruker SMART CCD area-detector diffractometer	4773 independent reflections
Radiation source: fine-focus sealed tube	1979 reflections with *I* > 2σ(*I*)
graphite	*R*_int_ = 0.036
φ and ω scans	θ_max_ = 25.0°, θ_min_ = 1.6°
Absorption correction: multi-scan (*SADABS*; Sheldrick, 1996)	*h* = −10→8
*T*_min_ = 0.961, *T*_max_ = 0.988	*k* = −14→14
7223 measured reflections	*l* = −15→16

### Refinement

**Table:**

Refinement on *F*^2^	Primary atom site location: structure-invariant direct methods
Least-squares matrix: full	Secondary atom site location: difference Fourier map
*R*[*F*^2^ > 2σ(*F*^2^)] = 0.066	Hydrogen site location: inferred from neighbouring sites
*wR*(*F*^2^) = 0.120	H-atom parameters constrained
*S* = 1.00	*w* = 1/[σ^2^(*F*_o_^2^) + (0.0296*P*)^2^] where *P* = (*F*_o_^2^ + 2*F*_c_^2^)/3
4773 reflections	(Δ/σ)_max_ < 0.001
352 parameters	Δρ_max_ = 0.16 e Å^−^^3^
0 restraints	Δρ_min_ = −0.18 e Å^−^^3^

### Special details

**Table:**

Geometry. All e.s.d.'s (except the e.s.d. in the dihedral angle between two l.s. planes) are estimated using the full covariance matrix. The cell e.s.d.'s are taken into account individually in the estimation of e.s.d.'s in distances, angles and torsion angles; correlations between e.s.d.'s in cell parameters are only used when they are defined by crystal symmetry. An approximate (isotropic) treatment of cell e.s.d.'s is used for estimating e.s.d.'s involving l.s. planes.
Refinement. Refinement of *F*^2^ against ALL reflections. The weighted *R*-factor *wR* and goodness of fit *S* are based on *F*^2^, conventional *R*-factors *R* are based on *F*, with *F* set to zero for negative *F*^2^. The threshold expression of *F*^2^ > σ(*F*^2^) is used only for calculating *R*-factors(gt) *etc*. and is not relevant to the choice of reflections for refinement. *R*-factors based on *F*^2^ are statistically about twice as large as those based on *F*, and *R*- factors based on ALL data will be even larger.

### Fractional atomic coordinates and isotropic or equivalent isotropic displacement parameters (Å^2^)

**Table:**

	*x*	*y*	*z*	*U*_iso_*/*U*_eq_	
F1	−0.6967 (3)	0.6507 (3)	0.4242 (2)	0.1682 (12)	
F2	0.5065 (3)	−0.0663 (2)	0.3998 (2)	0.1646 (12)	
O1	0.3825 (3)	0.4015 (2)	0.05479 (17)	0.0724 (7)	
O2	−0.1950 (3)	0.3213 (2)	0.1939 (2)	0.0921 (9)	
O3	−0.0319 (4)	0.1522 (3)	0.5289 (2)	0.1190 (11)	
O4	0.0495 (3)	0.5117 (2)	0.31565 (17)	0.0687 (7)	
O5	0.8805 (3)	0.9228 (2)	0.0997 (2)	0.0952 (8)	
H5	0.9547	0.9075	0.0620	0.143*	
N1	−0.1126 (3)	0.6500 (2)	0.1209 (2)	0.0631 (8)	
H1	−0.1678	0.7246	0.1130	0.076*	
N2	0.0779 (3)	0.7345 (2)	0.0419 (2)	0.0641 (8)	
N3	0.3234 (3)	0.6058 (3)	0.01820 (19)	0.0644 (8)	
H3	0.4169	0.6010	−0.0035	0.077*	
N4	0.2777 (3)	0.8125 (3)	−0.0313 (2)	0.0967 (11)	
H4A	0.2176	0.8855	−0.0376	0.116*	
H4B	0.3725	0.8012	−0.0519	0.116*	
N5	−0.1246 (3)	0.2430 (3)	0.3629 (3)	0.0754 (9)	
N6	0.0080 (3)	0.3332 (3)	0.4216 (2)	0.0633 (8)	
C1	0.0370 (4)	0.6286 (3)	0.0878 (2)	0.0542 (9)	
C2	0.2236 (5)	0.7165 (4)	0.0104 (3)	0.0666 (10)	
C3	0.2842 (4)	0.4988 (3)	0.0593 (2)	0.0572 (9)	
C4	0.1348 (4)	0.5102 (3)	0.1027 (2)	0.0511 (8)	
C5	0.0803 (3)	0.3989 (3)	0.1575 (2)	0.0534 (9)	
H5A	0.0389	0.3884	0.1031	0.064*	
C6	−0.0593 (3)	0.4266 (3)	0.2334 (2)	0.0508 (8)	
C7	−0.1839 (4)	0.5532 (3)	0.1689 (3)	0.0587 (9)	
H7	−0.2202	0.5436	0.1121	0.070*	
C8	−0.1336 (4)	0.3270 (3)	0.2608 (3)	0.0645 (10)	
C9	−0.0484 (5)	0.2372 (4)	0.4437 (3)	0.0788 (12)	
C10	0.0015 (4)	0.4300 (3)	0.3251 (3)	0.0551 (9)	
C11	−0.1894 (5)	0.1422 (4)	0.3883 (3)	0.1337 (18)	
H11A	−0.2645	0.1673	0.3358	0.201*	
H11B	−0.2387	0.1255	0.4557	0.201*	
H11C	−0.1073	0.0683	0.3900	0.201*	
C12	0.0864 (4)	0.3261 (4)	0.5089 (3)	0.0979 (13)	
H12A	0.0106	0.3622	0.5507	0.147*	
H12B	0.1590	0.3710	0.4813	0.147*	
H12C	0.1408	0.2406	0.5517	0.147*	
C13	0.2024 (4)	0.2751 (3)	0.2181 (3)	0.0566 (9)	
C14	0.2005 (5)	0.1674 (4)	0.2162 (3)	0.0923 (13)	
H14	0.1292	0.1720	0.1731	0.111*	
C15	0.3015 (6)	0.0531 (4)	0.2763 (4)	0.1288 (19)	
H15	0.2984	−0.0190	0.2744	0.155*	
C16	0.4049 (6)	0.0471 (4)	0.3381 (4)	0.1049 (15)	
C17	0.4125 (4)	0.1504 (4)	0.3425 (3)	0.0855 (12)	
H17	0.4847	0.1443	0.3858	0.103*	
C18	0.3109 (4)	0.2647 (3)	0.2815 (3)	0.0658 (10)	
H18	0.3160	0.3362	0.2834	0.079*	
C19	−0.3239 (4)	0.5881 (3)	0.2339 (3)	0.0614 (9)	
C20	−0.4521 (4)	0.5589 (3)	0.2302 (3)	0.0846 (12)	
H20	−0.4539	0.5245	0.1839	0.102*	
C21	−0.5790 (5)	0.5796 (4)	0.2940 (4)	0.1103 (16)	
H21	−0.6653	0.5586	0.2921	0.132*	
C22	−0.5731 (6)	0.6310 (5)	0.3588 (4)	0.1072 (16)	
C23	−0.4515 (5)	0.6654 (4)	0.3646 (3)	0.1005 (14)	
H23	−0.4530	0.7024	0.4096	0.121*	
C24	−0.3249 (4)	0.6426 (3)	0.3002 (3)	0.0776 (11)	
H24	−0.2395	0.6646	0.3021	0.093*	
C25	0.9345 (6)	0.8842 (4)	0.2021 (3)	0.1126 (15)	
H25A	0.9644	0.7943	0.2376	0.135*	
H25B	0.8507	0.9193	0.2416	0.135*	
C26	1.0640 (7)	0.9217 (5)	0.2010 (4)	0.168 (2)	
H26A	1.1452	0.8910	0.1588	0.251*	
H26B	1.1015	0.8884	0.2713	0.251*	
H26C	1.0323	1.0109	0.1720	0.251*	

### Atomic displacement parameters (Å^2^)

**Table:**

	*U*^11^	*U*^22^	*U*^33^	*U*^12^	*U*^13^	*U*^23^
F1	0.0895 (19)	0.212 (3)	0.191 (3)	−0.039 (2)	0.072 (2)	−0.101 (2)
F2	0.164 (3)	0.0856 (19)	0.182 (3)	0.0200 (18)	−0.073 (2)	−0.0244 (18)
N1	0.0516 (19)	0.0545 (17)	0.073 (2)	−0.0138 (16)	0.0088 (15)	−0.0226 (15)
N2	0.060 (2)	0.0560 (19)	0.0677 (19)	−0.0207 (17)	0.0147 (16)	−0.0208 (16)
N3	0.0527 (19)	0.0603 (19)	0.0658 (19)	−0.0183 (17)	0.0126 (15)	−0.0168 (16)
N4	0.083 (2)	0.067 (2)	0.123 (3)	−0.0329 (19)	0.026 (2)	−0.024 (2)
N5	0.076 (2)	0.069 (2)	0.085 (3)	−0.0421 (19)	0.011 (2)	−0.023 (2)
N6	0.071 (2)	0.067 (2)	0.0500 (19)	−0.0254 (17)	0.0058 (16)	−0.0221 (17)
O1	0.0607 (16)	0.0671 (16)	0.0791 (17)	−0.0149 (14)	0.0180 (13)	−0.0310 (13)
O2	0.087 (2)	0.0913 (19)	0.113 (2)	−0.0431 (16)	−0.0262 (17)	−0.0361 (17)
O3	0.171 (3)	0.085 (2)	0.075 (2)	−0.046 (2)	0.013 (2)	−0.0092 (17)
O4	0.0741 (17)	0.0823 (17)	0.0686 (16)	−0.0397 (15)	0.0053 (13)	−0.0379 (14)
O5	0.101 (2)	0.0892 (19)	0.0798 (19)	−0.0185 (16)	0.0004 (16)	−0.0310 (16)
C1	0.049 (2)	0.064 (2)	0.049 (2)	−0.018 (2)	0.0062 (17)	−0.0249 (18)
C2	0.069 (3)	0.054 (2)	0.063 (2)	−0.021 (2)	0.006 (2)	−0.013 (2)
C3	0.056 (3)	0.061 (2)	0.049 (2)	−0.014 (2)	0.0053 (18)	−0.0221 (19)
C4	0.048 (2)	0.049 (2)	0.053 (2)	−0.0141 (18)	0.0066 (17)	−0.0213 (17)
C5	0.052 (2)	0.059 (2)	0.054 (2)	−0.0166 (18)	0.0020 (17)	−0.0290 (18)
C6	0.044 (2)	0.057 (2)	0.056 (2)	−0.0205 (18)	−0.0025 (17)	−0.0224 (17)
C7	0.046 (2)	0.063 (2)	0.067 (2)	−0.0184 (19)	−0.0033 (19)	−0.0252 (19)
C8	0.046 (2)	0.067 (3)	0.082 (3)	−0.019 (2)	−0.004 (2)	−0.029 (2)
C9	0.088 (3)	0.068 (3)	0.063 (3)	−0.018 (3)	0.017 (3)	−0.021 (2)
C10	0.040 (2)	0.066 (2)	0.059 (2)	−0.0154 (19)	0.0091 (18)	−0.030 (2)
C11	0.154 (4)	0.114 (4)	0.155 (5)	−0.098 (4)	0.020 (4)	−0.037 (3)
C12	0.126 (4)	0.107 (3)	0.057 (3)	−0.029 (3)	−0.014 (3)	−0.034 (2)
C13	0.052 (2)	0.063 (2)	0.054 (2)	−0.016 (2)	0.0027 (18)	−0.0261 (18)
C14	0.103 (3)	0.058 (3)	0.117 (4)	−0.009 (3)	−0.029 (3)	−0.041 (3)
C15	0.140 (5)	0.065 (3)	0.173 (5)	−0.007 (3)	−0.055 (4)	−0.045 (3)
C16	0.106 (4)	0.059 (3)	0.112 (4)	0.005 (3)	−0.023 (3)	−0.019 (3)
C17	0.067 (3)	0.089 (3)	0.082 (3)	−0.009 (3)	−0.018 (2)	−0.026 (3)
C18	0.057 (2)	0.063 (2)	0.069 (2)	−0.013 (2)	0.000 (2)	−0.024 (2)
C19	0.037 (2)	0.071 (2)	0.071 (3)	−0.0140 (19)	0.0028 (19)	−0.028 (2)
C20	0.044 (2)	0.100 (3)	0.117 (3)	−0.024 (2)	0.006 (2)	−0.052 (3)
C21	0.055 (3)	0.125 (4)	0.156 (5)	−0.038 (3)	0.023 (3)	−0.063 (4)
C22	0.067 (4)	0.120 (4)	0.125 (4)	−0.027 (3)	0.042 (3)	−0.057 (3)
C23	0.073 (3)	0.120 (4)	0.117 (4)	−0.022 (3)	0.019 (3)	−0.069 (3)
C24	0.055 (3)	0.097 (3)	0.093 (3)	−0.022 (2)	0.016 (2)	−0.057 (3)
C25	0.147 (5)	0.107 (4)	0.082 (3)	−0.052 (3)	0.000 (3)	−0.028 (3)
C26	0.210 (7)	0.201 (6)	0.124 (4)	−0.119 (6)	−0.008 (4)	−0.051 (4)

### Geometric parameters (Å, °)

**Table:**

F1—C22	1.376 (5)	C7—C19	1.511 (4)
F2—C16	1.376 (4)	C7—H7	0.9800
N1—C1	1.357 (4)	C11—H11A	0.9600
N1—C7	1.453 (3)	C11—H11B	0.9600
N1—H1	0.8600	C11—H11C	0.9600
N2—C2	1.320 (4)	C12—H12A	0.9600
N2—C1	1.374 (4)	C12—H12B	0.9600
N3—C2	1.335 (4)	C12—H12C	0.9600
N3—C3	1.378 (4)	C13—C18	1.374 (4)
N3—H3	0.8600	C13—C14	1.375 (4)
N4—C2	1.338 (4)	C14—C15	1.373 (5)
N4—H4A	0.8600	C14—H14	0.9300
N4—H4B	0.8600	C15—C16	1.346 (5)
N5—C8	1.366 (4)	C15—H15	0.9300
N5—C9	1.388 (4)	C16—C17	1.355 (5)
N5—C11	1.478 (4)	C17—C18	1.379 (4)
N6—C10	1.375 (4)	C17—H17	0.9300
N6—C9	1.382 (4)	C18—H18	0.9300
N6—C12	1.474 (4)	C19—C20	1.372 (4)
O1—C3	1.258 (3)	C19—C24	1.374 (4)
O2—C8	1.209 (4)	C20—C21	1.384 (5)
O3—C9	1.203 (4)	C20—H20	0.9300
O4—C10	1.210 (3)	C21—C22	1.343 (6)
O5—C25	1.418 (4)	C21—H21	0.9300
O5—H5	0.8200	C22—C23	1.363 (5)
C1—C4	1.380 (4)	C23—C24	1.392 (5)
C3—C4	1.406 (4)	C23—H23	0.9300
C4—C5	1.508 (4)	C24—H24	0.9300
C5—C13	1.522 (4)	C25—C26	1.428 (5)
C5—C6	1.585 (4)	C25—H25A	0.9700
C5—H5A	0.9800	C25—H25B	0.9700
C6—C10	1.507 (4)	C26—H26A	0.9600
C6—C8	1.521 (4)	C26—H26B	0.9600
C6—C7	1.564 (4)	C26—H26C	0.9600
			
C1—N1—C7	123.3 (3)	H11A—C11—H11B	109.5
C1—N1—H1	118.3	N5—C11—H11C	109.5
C7—N1—H1	118.3	H11A—C11—H11C	109.5
C2—N2—C1	114.7 (3)	H11B—C11—H11C	109.5
C2—N3—C3	123.3 (3)	N6—C12—H12A	109.5
C2—N3—H3	118.4	N6—C12—H12B	109.5
C3—N3—H3	118.4	H12A—C12—H12B	109.5
C2—N4—H4A	120.0	N6—C12—H12C	109.5
C2—N4—H4B	120.0	H12A—C12—H12C	109.5
H4A—N4—H4B	120.0	H12B—C12—H12C	109.5
C8—N5—C9	125.8 (3)	C18—C13—C14	117.5 (3)
C8—N5—C11	117.4 (3)	C18—C13—C5	122.3 (3)
C9—N5—C11	116.5 (4)	C14—C13—C5	120.1 (3)
C10—N6—C9	125.3 (3)	C15—C14—C13	121.6 (4)
C10—N6—C12	118.7 (3)	C15—C14—H14	119.2
C9—N6—C12	116.0 (3)	C13—C14—H14	119.2
C25—O5—H5	109.5	C16—C15—C14	118.9 (4)
N1—C1—N2	113.2 (3)	C16—C15—H15	120.5
N1—C1—C4	121.5 (3)	C14—C15—H15	120.5
N2—C1—C4	125.3 (3)	C15—C16—C17	122.0 (4)
N2—C2—N3	123.5 (3)	C15—C16—F2	120.1 (5)
N2—C2—N4	119.7 (4)	C17—C16—F2	117.9 (5)
N3—C2—N4	116.8 (4)	C16—C17—C18	118.6 (4)
O1—C3—N3	118.3 (3)	C16—C17—H17	120.7
O1—C3—C4	126.1 (3)	C18—C17—H17	120.7
N3—C3—C4	115.6 (3)	C13—C18—C17	121.4 (4)
C1—C4—C3	117.1 (3)	C13—C18—H18	119.3
C1—C4—C5	121.4 (3)	C17—C18—H18	119.3
C3—C4—C5	121.4 (3)	C20—C19—C24	118.7 (3)
C4—C5—C13	116.7 (3)	C20—C19—C7	118.7 (4)
C4—C5—C6	109.9 (3)	C24—C19—C7	122.5 (3)
C13—C5—C6	109.1 (2)	C19—C20—C21	121.3 (4)
C4—C5—H5A	106.9	C19—C20—H20	119.3
C13—C5—H5A	106.9	C21—C20—H20	119.3
C6—C5—H5A	106.9	C22—C21—C20	117.6 (4)
C10—C6—C8	115.5 (3)	C22—C21—H21	121.2
C10—C6—C7	111.9 (3)	C20—C21—H21	121.2
C8—C6—C7	106.2 (3)	C21—C22—C23	124.2 (4)
C10—C6—C5	109.1 (2)	C21—C22—F1	118.7 (5)
C8—C6—C5	105.7 (3)	C23—C22—F1	117.0 (5)
C7—C6—C5	108.1 (2)	C22—C23—C24	117.0 (4)
N1—C7—C19	112.1 (3)	C22—C23—H23	121.5
N1—C7—C6	109.7 (2)	C24—C23—H23	121.5
C19—C7—C6	112.5 (3)	C19—C24—C23	121.1 (4)
N1—C7—H7	107.4	C19—C24—H24	119.5
C19—C7—H7	107.4	C23—C24—H24	119.5
C6—C7—H7	107.4	O5—C25—C26	112.3 (4)
O2—C8—N5	121.6 (3)	O5—C25—H25A	109.1
O2—C8—C6	120.8 (4)	C26—C25—H25A	109.1
N5—C8—C6	117.5 (3)	O5—C25—H25B	109.1
O3—C9—N6	121.7 (4)	C26—C25—H25B	109.1
O3—C9—N5	121.5 (4)	H25A—C25—H25B	107.9
N6—C9—N5	116.9 (4)	C25—C26—H26A	109.5
O4—C10—N6	119.6 (3)	C25—C26—H26B	109.5
O4—C10—C6	122.3 (3)	H26A—C26—H26B	109.5
N6—C10—C6	118.0 (3)	C25—C26—H26C	109.5
N5—C11—H11A	109.5	H26A—C26—H26C	109.5
N5—C11—H11B	109.5	H26B—C26—H26C	109.5
			
C7—N1—C1—N2	178.1 (3)	C10—N6—C9—O3	176.3 (3)
C7—N1—C1—C4	−3.3 (5)	C12—N6—C9—O3	−1.1 (5)
C2—N2—C1—N1	178.9 (3)	C10—N6—C9—N5	−3.5 (5)
C2—N2—C1—C4	0.4 (5)	C12—N6—C9—N5	179.1 (3)
C1—N2—C2—N3	2.5 (5)	C8—N5—C9—O3	−171.9 (4)
C1—N2—C2—N4	−177.2 (3)	C11—N5—C9—O3	2.4 (6)
C3—N3—C2—N2	0.5 (5)	C8—N5—C9—N6	7.8 (5)
C3—N3—C2—N4	−179.8 (3)	C11—N5—C9—N6	−177.9 (3)
C2—N3—C3—O1	173.9 (3)	C9—N6—C10—O4	177.2 (3)
C2—N3—C3—C4	−6.0 (5)	C12—N6—C10—O4	−5.5 (5)
N1—C1—C4—C3	175.8 (3)	C9—N6—C10—C6	−5.5 (5)
N2—C1—C4—C3	−5.8 (5)	C12—N6—C10—C6	171.9 (3)
N1—C1—C4—C5	−0.6 (5)	C8—C6—C10—O4	−172.6 (3)
N2—C1—C4—C5	177.8 (3)	C7—C6—C10—O4	−51.0 (4)
O1—C3—C4—C1	−171.8 (3)	C5—C6—C10—O4	68.6 (4)
N3—C3—C4—C1	8.1 (4)	C8—C6—C10—N6	10.1 (4)
O1—C3—C4—C5	4.6 (5)	C7—C6—C10—N6	131.7 (3)
N3—C3—C4—C5	−175.5 (3)	C5—C6—C10—N6	−108.7 (3)
C1—C4—C5—C13	−149.9 (3)	C4—C5—C13—C18	43.5 (4)
C3—C4—C5—C13	33.9 (4)	C6—C5—C13—C18	−81.7 (4)
C1—C4—C5—C6	−25.0 (4)	C4—C5—C13—C14	−140.8 (3)
C3—C4—C5—C6	158.7 (3)	C6—C5—C13—C14	94.0 (4)
C4—C5—C6—C10	−70.3 (3)	C18—C13—C14—C15	0.9 (6)
C13—C5—C6—C10	58.8 (3)	C5—C13—C14—C15	−174.9 (4)
C4—C5—C6—C8	164.9 (3)	C13—C14—C15—C16	−0.3 (7)
C13—C5—C6—C8	−66.0 (3)	C14—C15—C16—C17	−0.1 (8)
C4—C5—C6—C7	51.5 (3)	C14—C15—C16—F2	179.5 (4)
C13—C5—C6—C7	−179.4 (3)	C15—C16—C17—C18	−0.1 (7)
C1—N1—C7—C19	158.4 (3)	F2—C16—C17—C18	−179.7 (3)
C1—N1—C7—C6	32.6 (4)	C14—C13—C18—C17	−1.2 (5)
C10—C6—C7—N1	65.0 (3)	C5—C13—C18—C17	174.6 (3)
C8—C6—C7—N1	−168.2 (3)	C16—C17—C18—C13	0.8 (6)
C5—C6—C7—N1	−55.2 (3)	N1—C7—C19—C20	139.9 (3)
C10—C6—C7—C19	−60.5 (4)	C6—C7—C19—C20	−95.8 (4)
C8—C6—C7—C19	66.3 (4)	N1—C7—C19—C24	−43.2 (4)
C5—C6—C7—C19	179.3 (3)	C6—C7—C19—C24	81.1 (4)
C9—N5—C8—O2	175.8 (4)	C24—C19—C20—C21	−2.1 (6)
C11—N5—C8—O2	1.5 (5)	C7—C19—C20—C21	174.9 (4)
C9—N5—C8—C6	−2.5 (5)	C19—C20—C21—C22	0.9 (7)
C11—N5—C8—C6	−176.8 (3)	C20—C21—C22—C23	0.9 (8)
C10—C6—C8—O2	175.2 (3)	C20—C21—C22—F1	−179.0 (4)
C7—C6—C8—O2	50.6 (4)	C21—C22—C23—C24	−1.4 (7)
C5—C6—C8—O2	−64.1 (4)	F1—C22—C23—C24	178.5 (4)
C10—C6—C8—N5	−6.4 (4)	C20—C19—C24—C23	1.6 (5)
C7—C6—C8—N5	−131.0 (3)	C7—C19—C24—C23	−175.4 (3)
C5—C6—C8—N5	114.3 (3)	C22—C23—C24—C19	0.2 (6)

### Hydrogen-bond geometry (Å, °)

**Table:**

*D*—H···*A*	*D*—H	H···*A*	*D*···*A*	*D*—H···*A*
N3—H3···O1^i^	0.86	1.88	2.737 (3)	177
N4—H4A···O5^ii^	0.86	2.07	2.890 (3)	160
O5—H5···N2^iii^	0.82	2.19	2.779 (3)	129

Symmetry codes: (i) −*x*+1, −*y*+1, −*z*; (ii) −*x*+1, −*y*+2, −*z*; (iii) *x*+1, *y*, *z*.
